# Biological Activities and Cytotoxicity of Diterpenes from *Copaifera* spp. Oleoresins

**DOI:** 10.3390/molecules20046194

**Published:** 2015-04-09

**Authors:** Fabiano de S. Vargas, Patrícia D. O. de Almeida, Elenn Suzany P. Aranha, Ana Paula de A. Boleti, Peter Newton, Marne C. de Vasconcellos, Valdir F. Veiga Junior, Emerson S. Lima

**Affiliations:** 1 Chemistry Department, Federal University of Amazonas, Av. Gal. Rodrigo Octávio, 6.200, Japiim, Manaus-AM 69080-900, Brazil; E-Mails: fabianosvargas@gmail.com (F.S.V.); valdirveiga@ufam.edu.br (V.F.V.J.); 2 Faculty of Pharmaceutical Sciences, Federal University of Amazonas, Manaus, Av. Gal. Rodrigo Octávio, 6.200, Japiim, Manaus-AM 69080-900, Brazil; E-Mails: patt_danielle@hotmail.com (P.D.O.A.); marnevasconcellos@yahoo.com.br (E.S.P.A.); apboleti@yahoo.com.br (A.P.A.B.); marne@ufam.edu.br (M.C.V.); 3 Environmental Studies Program, University of Colorado, Boulder, CO 80309, USA; E-Mail: peter.newton@colorado.edu; 4 School of Environmental Sciences, University of East Anglia, Norwich NR4 7TJ, UK

**Keywords:** *Copaifera* L., diterpenic acids, anti-inflammatory, antitumoral

## Abstract

*Copaifera* spp. are Amazonian species widely studied and whose oleoresins are used by local people for various medicinal purposes. However, a detailed study of the activity of the main phytochemical components of these oleoresins remains to be done. Here, we studied the cytotoxicity and *in vitro* anti-inflammatory effects of six diterpene acids: copalic, 3-hydroxy-copalic, 3-acetoxy-copalic, hardwickiic, kolavic-15-metyl ester, and kaurenoic, isolated from the oleoresins of *Copaifera* spp. The diterpenes did not show cytotoxicity in normal cell lines, nor did they show significant changes in viability of tumoral line cells. The 3-hydroxy-copalic was able to inhibit the enzyme tyrosinase (64% ± 1.5%) at 250 µM. The kolavic-15-metyl ester at 200 µM showed high inhibitory effect on lipoxygenase (89.5% ± 1.2%). Among the diterpenes tested, only kaurenoic and copalic acids showed significant hemolytic activities with 61.7% and 38.4% at 100 µM, respectively. In addition, it was observed that only the copalic acid (98.5% ± 1.3%) and hardwickiic acid (92.7% ± 4.9%) at 100 mM inhibited nitric oxide production in macrophages activated by lipopolysaccharide. In this assay, the diterpenes did not inhibit tumor necrosis factor-α production. The acids inhibited the production of IL-6, 3-acetoxy-copalic (23.8% ± 8.2%), kaurenoic (11.2% ± 5.7%), kolavic-15-methyl ester (17.3% ± 4.2%), and copalic (4.2% ± 1.8%), respectively, at 25 µM. The kaurenoic, 3-acetoxy-copalic and copalic acids increased IL-10 production. This study may provide a basis for future studies on the therapeutic role of diterpenic acids in treating acute injuries such as inflammation or skin disorders.

## 1. Introduction

*Copaifera* species, known in Brazil as “copaíbeiras” or “copaíba”, are Amazonian plants best known and used by traditional Amazonian people for the pharmacological activity of the oleoresins that they contain. The species of the *Copaifera* genus are among the most studied in the world, due to their economic and ecological importance. *Copaíba* oleoresin is exuded from the tree trunks of certain *Copaifera* species and has been demonstrated to be primarily composed of sesquiterpenes and diterpenes. The oleoresin has widespread cosmetic and medicinal purposes due to its emollient, bactericide, anti-inflammatory, and anti-melasma properties [1,2]. Ten species found in Brazil have been studied for their oleoresin, which has resulted in more than 100 sesquiterpenes and 40 diterpenes being identified [2,3]. However, few studies have isolated and described their biological activities.

The chemical composition of *Copaifera* spp. oleoresin has been well established in several studies, and the oleoresin is defined as a mixture of a predominant fraction of sesquiterpenes and diterpenes, such as copalic acid, which is considered a biomarker for the oleoresin of copaíba [1,4]. Diterpenes such as abietic acid and acids of the raw resin have been subject of studies for over a century, but a complete understanding of the chemistry of diterpenes was developed only in the last 20 years. Some diterpenes are well known, such as Taxol (paclitaxel), which is used to treat breast cancer, stevioside from *Stevia rebaudiana* Bertoni (Asteraceae), the ginkgolides of medicinal plant *Ginkgo biloba* L., as well as resinous acids of coniferous or *Copaifera* spp. trees [5,6].

The pharmacological use of *Copaifera* spp. oleoresin as a homeopathic anti-inflammatory, antitumoral, and antibiotic agent is widespread [1,7,8]. The main compounds present in *Copaifera* spp. oleoresin have been widely described in the literature, yet despite pronounced biological activities described among the diverse substances identified in *Copaifera* spp. oleoresins, few have been isolated and analyzed on a larger scale with respect to their biological activities [2]. Rich fractions of sesquiterpenes are well known for having important anti-inflammatory effects, in addition to having probable synergistic effects, especially antimicrobial and antileishmanial, analgesic and antitumoral activity [9]. However, these activities alone do not explain the strong pharmacological activity of *Copaifera* spp. oleoresin. Some studies have reported that increased anti-inflammatory activity can be correlated with the presence of high levels of diterpene acids [10,11]. 

Based on these studies, and in order to fill some critical gaps in our knowledge of *Copaifera* spp. oleoresin, this study aimed to detect and isolate diterpenic acids of *Copaifera* spp. oloeresin on a gram-scale and evaluate their *in vitro* anti-inflammatory effects using a J774 macrophage model and cytotoxicity in normal and tumoral cells.

## 2. Results and Discussion

### 2.1. Inhibition of Tyrosinase

Tyrosinase is an enzyme that triggers melanogenesis, initiating a cascade of reactions that convert tyrosine to the biopolymer melanin for mechanisms of enzymatic oxidation [12,13,14]. Thus, the assay for determining inhibition of tyrosinase reveals an important system for eliminating radical species [15,16], so that the compounds with antioxidant potential capable of inhibiting the production of free radicals may reduce or prevent hyperpigmentation melanogenesis. Thus, the compounds capable of inhibiting the enzyme tyrosinase can be considered excellent candidates for developing new skin bleaching agents as well as for treating hyperpigmentation or medical conditions such as melasma and post-inflammatory melanoderm.

**Figure 1:**
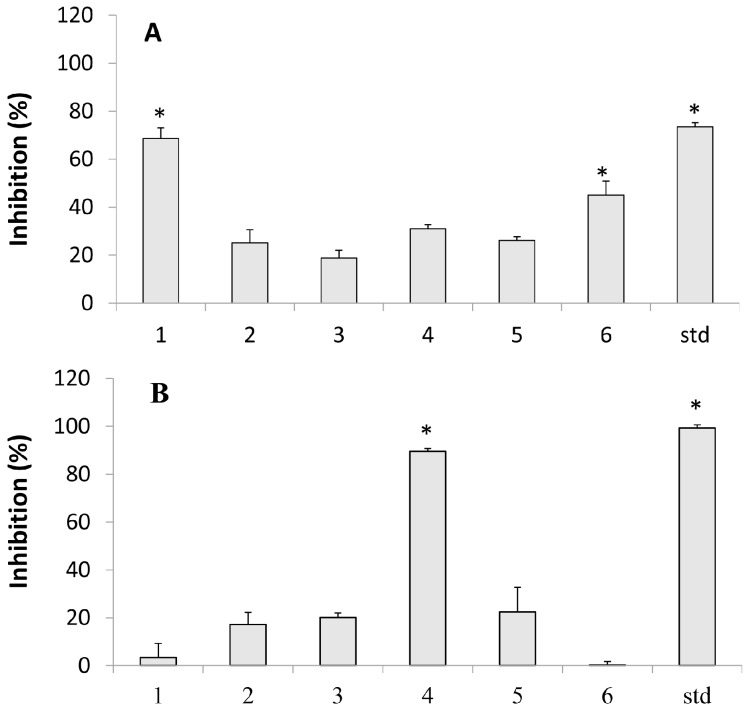
Tyrosinase and lipoxygenase activities of the isolated diterpenes from *Copaifera* sp. (**A**) Tyrosinase—Samples at 250 µM, and Kojic acid as standard (std); (**B**) Lipoxygenase—Samples at 200 µM, and quercertin as standard (std). 1. 3-hydroxy-copalic acid, 2. kaurenoic acid, 3. 3-acetoxy-copalic acid, 4. kolavic acid15-methyl ester, 5. copalic acid, and 6. hardwickiic acid. Each value is the mean ± S.E. of three independent experiments. Statistical analysis was performed using Holm-Sidak multiple comparisons. * *p* < 0.05 compared with the negative control.

The tyrosinase inhibition test was performed at concentrations of 31.25 to 500 µM. It was observed that all diterpenes showed inhibition below 20%, except 3-hydroxy-copalic that showed a greater capacity for this enzyme inhibition with IC_50_ of 255.5 µM. Kojic acid, the positive control, showed IC_50_ of 211.2 µM (Figure 1).

There are few studies that report diterpenic acids showing inhibitory activity for tyrosinase. Ko *et al.* [17] reported three new *ent*-kaurane-type diterpenes isolated from leaves of *Broussonetia papyrifera*, which were effective as mushroom tyrosinase inhibitors. In addition, methanolic extract from leaves of this plant displayed a greater mushroom tyrosinase inhibitory effect (IC_50_ = 104.2 μg/mL) when compared with kojic acid (IC_50_ = 500 μg/mL). The activity of *ent*-kauranes could be due to the 6-oxo-7-hydroxy carbinol moiety due to the structural similarity to kojic acid and tropolone, the most potent tyrosinase inhibitor [17].

Similarly, Oliveira *et al.* [18] evaluated the *in vitro* effect of copaíba oleoresin with kojic acid, using a snakeskin model. It was observed that solutions containing copaíba oleoresin at 25% and 50% induced kojic acid penetration in 4.0 and 3.7, respectively. The author concluded that this oleoresin has the potential to be added in topical formulations as a promoter of penetration to hydrophilic substances and to contribute to the development of more effective topical depigmenting agents. In our assay, kojic acid was used as a standard with activity of 73.5% at 700 µM. The 3-hydroxy-copalic showed activity of 63.9% at a concentration of 250 µM. Thus, 3-hydroxy-copalic was three times more active than the standard used, showing a strong potential to inhibit melanogenesis.

### 2.2. Lipoxygenase Inhibition

Lipoxygenase (LOX) is a key enzyme that catalyzes the specific regions oxygenated by polyunsaturated fatty acids. By adding oxygen molecules, it transforms linoleic and arachidonic acid into hydroperoxide compounds. This metabolism of arachidonic acid by lipoxygenase generates several biologically active lipids that play important roles in the inflammation process [19,20,21].

Research on new inhibitors of LOX therefore has potential benefits not only for treatment of inflammation, but also for the treatment of a variety of other diseases such as strokes, arthritis, asthma, and tumor inhibition [22]. Figure 1 shows the effects of diterpenes on inhibiting LOX. Our results showed that only kolavic acid -15-methyl ester exhibited a significant inhibition with IC_50_ of 157.8 ± 17.7 µM. This result was not as effective as standard quercetin used here as standard, with IC_50_ = 30.2 ± 0.6 µM. In recent studies, Khan *et al.* [23] isolated two abietane diterpenes from species *Taxus wallichiana* Zucc, taxusabietane C (TC) and taxamairin F (TF), which showed an inhibitory effect on LOX, with IC_50_ values of 69 ± 0.31 and 73 ± 0.14 µM, respectively. Abe *et al.* [24] evaluated the activity of two new labdane diterpenes on the LOX enzyme, which showed strong inhibitory activity with IC_50_ of 7.5 µM and 4.0 µM.

### 2.3. Hemolytic Activity

The assay for the characterization of hemolytic activity aimed to evaluate the potential of diterpenes on lysis of the cell membrane. The absence of hemolysis is indicative that the diterpenes can be tolerated by the biological system. Among the acids tested, none showed significant hemolytic activity in concentrations between 5 and 50 mM. However, kaurenoic and copalic acids showed significant hemolytic activity of 61.7% and 38.4% at 100 mM, with IC_50_ of 86.7 ± 4.1 mM and 169.8 ± 11.8 mM, respectively. Similarly, Costa-Lotufo *et al.* [25] evaluated the hemolytic potential of kaurenoic acid in erythrocytes of rats and humans, with IC_50_ of 74 mM and 56.4 mM, respectively. They confirmed that the compound destabilized the membrane causing hemolysis compatible with increasing concentrations, which would reflect its cytotoxicity by nonspecific mechanisms.

### 2.4. Cytotoxicity

Prior to the tests in cell culture, cytotoxicity was evaluated to determine toxic and nontoxic concentrations to be tested in subsequent assays. We evaluated the capacity of six diterpenic acids (kaurenoic, copalic, 3 -hydroxy-copalic, kolavic-15-methyl ester, 3-acetoxy-copalic, and hardwickiic) to interfere with the murine fibroblast 3T3-L1, human lymphocytes, and J774 murine macrophage viability. In tests with 3T3-L1 cells, it was observed that the compounds tested at 5 mg/mL showed no significant reduction in viability after 24 hours of treatment. This result is similar to the result obtained by Masson-Myers *et al.* [26] showing that 3T3 cells were viable at concentrations above 100 mg/mL in *C. langsdorffi* oleoresin. The diterpenes also were tested in a normal human lymphocyte line at concentrations of 7.8 to 500 µM, showing no significant reduction of cell viability. When determined whether diterpenes promoted cytotoxic effects on J774 macrophage our results showed that it did not produce a cytotoxic effect in normal cells, with IC_50_ below 100 µM.

We also investigated possible cytotoxic effects on tumor cell lines (Table 1). To characterize this activity, the compounds were tested in different tumor cell lines, among which were AGP-01 (gastric), HCT116 (colorectal), MCF07 (breast cancer), NIHOVCAR (ovary), SKAMELL-4 (melanoma), and SF-295 (human glioblastoma), each at a concentration of 20 µM. In the AGP01, HCT-116, and NIHOVCAR lines, the kolavic-15- methyl ester, kaurenoic, copalic, 3-HO-copalic, and 3-AcO-copalic acids showed low cytotoxicity. Only the SF-295 line was more sensitive: the kaurenoic diterpenes 3-HO-copalic and 3-AcO-copalic acids showed cytotoxicity of around 20%. Diterpenes did not alter cell viability, nor were they cytotoxic to the MCF07 and SKAMELL 4 lines.

**Table 1:** Viability inhibition (%) of diterpenes from *Copaifera* spp. in tumoral line cells after 72 h of exposition at 20 µmol/L.

	Kolavic Acid 15 Methyl Ester	Copalic Acid	Hardwickiic Acid	Kaurenoic Acid	3-Hydroxy-Copalic Acid	3-Acetoxy-Copalic Acid
**AGP01**	17.4 ± 6.7	NT	NT	28.1 ± 4.3	8.5 ± 13.1	13.1 ± 5.3
**HCT-116**	≤0.01	≤0.01	≤0.01	0.27 ± 4.3	4.7 ± 5.6	≤0.01
**NIHOVCAR**	≤0.01	≤0.01	≤0.01	≤0.01	≤0.01	2.0 ± 3.5
**SF295**	≤0.01	≤0.01	≤0.01	28.0 ± 6.5	20.5 ± 5.0	17.7 ± 0.9
**MCF7**	≤0.01	≤0.01	≤0.01	≤0.01	≤0.01	≤0.01
**SKMELL28**	≤0.01	≤0.01	≤0.01	≤0.01	≤0.01	≤0.01

NT = not tested.

There are few studies in the literature on diterpene acids in a tumoral line. Costa-Lotufo *et al.* [25] showed the effects of kaurenoic acid isolated from the oleoresin *Copaifera langsdorffi* in inhibiting tumor cell lines CEM (leukemia) by 95%, MCF -7 (breast cancer) by 45%, and HCT -8 cells (colon cancer) by 45%, in a single concentration of 78 µM. Zhao *et al.* [27] evaluated the cytotoxicity of clerodane diterpenes isolated from *Polyalthia longifolia*, including kolavic acid, on the lines A-549 (lung carcinoma), MCF-7 (breast cancer), and HT-29 (colon cancer). Gonzáles *et al.* [28] also showed the activity of sclareolide labdane derivatives against human cervical carcinoma cells (HELA) and normal monkey kidney cells (VERO), with expressive activities for the carcinogenic line at IC_50_ = 2.6 ± 0.2 mg/mL. Sashidhara *et al.* [29] showed cytotoxicity effects of clerodane diterpenes against ovarian carcinoma cell lines (PA1), breast adenocarcinoma (MCF-7), oral carcinoma (KB), and cervical carcinoma (C33A). All diterpenes showed moderate activity against the four cancer cell lines tested, with the most active compound showing IC_50_ = 20 mg/mL (±60 µM).

### 2.5. Inhibition of Nitric Oxide Production

Nitric oxide (NO^•^) produced by inducible nitric*-*oxide synthase (iNOS) is an important mediator of inflammation, but the excessive production of NO^•^ is highly associated with serious diseases such as septic shock, arthritis, stroke, and inflammatory, chronic, and autoimmune diseases. The determination of the rate of production of NO^•^ and iNOS protein expression in LPS stimulated cellular models has been a very useful tool in the search for compounds with anti-inflammatory properties [30].

**Figure 2:**
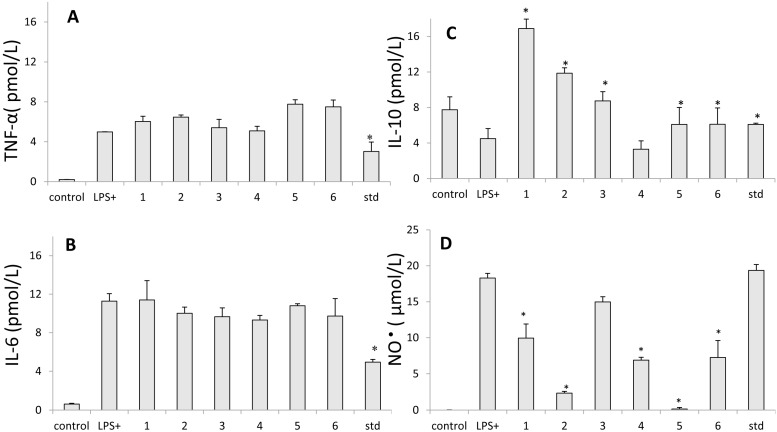
Effect of the isolated diterpenic acids on nitric oxide (NO^•^) and cytokine production in lipopolysaccharide-stimulated J774 cells. (**A**) TNF-α, (**B**) IL-6 and (**C**) IL-10 and (**D**) NO^•^. Production of NO^•^ was assayed in culture supernatants of macrophages stimulated with LPS (1 µg/mL) for 24 h in the presence of the four compounds (25 µg/mL to cytokines and 50 µg/mL to NO^•^). The nitrite and cytokines values are the mean ± S.D. from three independent experiments. Indomethacin was used as a standard. Statistical analysis was performed using the Holm-Sidak multiple comparison. * *p* < 0.05 when compared to control stimulated with LPS. (**1**) 3-hydroxy-copalic acid, (**2**) kaurenoic acid, (**3**) 3-acetoxy-copalic acid, (**4**) kolavic acid 15-methyl ester, (**5**) copalic acid and (**6**) hardwickiic acid.

Our study evaluated the effects of diterpene compounds on the production of nitric oxide in the murine macrophage cell line J774 stimulated with LPS (Figure 2D). NO^•^ levels were measured by the Greiss method. We confirmed that diterpenes in a concentration of 100 mM inhibited NO^•^ production in 90%, presenting IC_50_ = 57.4 ± 0.2 µM (copalic acid); 67.4 ± 1.3 µM (hardwickiic acid); 61.2 ± 4.2 µM (kaurenoic acid); 81.6 ± 2.8 µM (kolavic acid -15-methyl ester). The percentages of inhibition by copalic and kaurenoic acids were compared with the indomethacin, used as a standard, with IC_50_ = 32.0 ± 3.5 µM. Similar results for kaurenoic acid were observed by Choi *et al.* [31], who observed that the kaurenoic acid isolated from *Aralia continentalis* presented an inhibition with IC_50_ = 51.7 ± 2.4 µM in NO^•^ production in RAW 264.7 macrophage stimulated by LPS.

Many of the studies published on copaíba use raw oleoresin to evaluate anti-inflammatory activities A recent study observed that the oleoresin *C. multijuga* Hayne and *C. cearensis* ex Huber Ducke significantly inhibited NO^•^ production in mouse peritoneal macrophages stimulated by LPS by about 30% at a concentration of 50 mg/mL [32]. The oleoresin of *C. reticulata* was able to inhibit NO^•^ production by 85% at a concentration of 500 mg/mL. According to [32], the oleoresin of *C. multijuga* Hayne was the most potent *Copaifera* species and was able to inhibit the production of NO^•^ in the lowest concentration (5 mg/mL), whereas the oleoresin of *C. cearensis* ex Huber Ducke was only effective at 50 mg/mL and *C. reticulata* Ducke at 500 mg/mL. In contrast, Soares *et al.* [33] evaluated commercially-available *Copaifera* oleoresins and from one of these oils, fractions of sesquiterpenes and diterpenes were obtained, which at 10 mg/mL did not inhibit NO^•^ production in murine peritoneal macrophages stimulated by LPS.

In a similar study, Giron *et al.* [34] evaluated a series of eleven labdane diterpenes: two naturally occurring (galeopsin and hispanolone), and nine derivatives with potential anti-inflammatory activity, verifying a significant reduction in the NO^•^ and TNF-α production in RAW 264.7 macrophages activated by LPS (IC_50_ between 1 and 10 mM). Anti-inflammatory activity has been related to the inhibition of the expression of iNOS and cyclooxygenase-2 (COX-2) at the level of transcription, as determined by Western blot analysis and real-time PCR. Finally, Girón *et al.* [34] showed a series of nine labdane diterpenes derived from labdanodiol with anti-inflammatory potential in RAW 264.7 macrophages treated with LPS, in which all compounds also strongly inhibited the NO^•^ production, with IC_50_ from 5 to 15 µM; however, some derivatives showed moderate cytotoxic activity on macrophages.

### 2.6. Assay for Cytokine Production

Macrophages are an important source of many cytokines involved in the immune response, hematopoiesis, inflammation, and other homeostatic processes. After stimulation by microorganisms, microbial products (e.g., LPS), or endogenous factors (including cytokines), the macrophages synthesize and release a variety of cytokines: IL-1, IL-3, IL-4, IL-5, IL-6, IL-8, IL-9, IL-10, IL-12, IL-13, IL-17, TNF-α, IFN-α, IFN-γ, TGF-β, M-CSF, G-CSF, GM-CSF, MCP-1, MCP-3, MCP-5, MIP-1, MIP-2, RANTES, MIF and KC. Furthermore, these cytokines can modulate most functions of macrophages, by the expression of markers on the cell surface, cytokine secretion, and other mechanisms [35].

In this study, we first investigated the influence of isolated compounds of diterpene acids on the production of TNF-α, IL-6, and IL-10 (Figure 2A–C). The diterpenic showed no significant inhibition to TNF-α production at concentrations of 12.5 µM to 50 µM. These results are consistent with a study that evaluated anti-inflammatory activity of a purified fraction of *C. langsdorffi* containing diterpenic acids, sesquiterpenes, and diterpenes (named fraction ORPF) [3]. It was observed that ORPF significantly inhibited the production of TNF-α only at concentrations of 1 µM to 10 µM, showing no significant inhibition at concentrations less than or greater than these, and that this inhibition may have been associated with the presence of the sesquiterpene fraction studied [3]. In contrast, Diaz-Viciedo *et al.* [36] reported on the inhibitory activity on the production and expression of TNF-α exerted by kaurenoic acid, isolated from *Helianthus annuus* L., at concentrations ranging from 1 to 20 mg/mL. Castrillo *et al.* [37] also examined the ability of other diterpenes to exert inhibitory effects on the production of TNF-α, and evaluated the influence of six diterpenes, three kauranes (foliol, linearol acid, and *ent*-kaurenoic acid), and three clerodanes (teucrium A, acetilgnaphalin, and eriocephalin). Only kauranes showed inhibitory activity on the production of TNF-α.

Interleukin 6 (IL-6) plays a key role in the transition between acute inflammation and chronic inflammation, altering the nature of leukocyte infiltration (polymorphonuclear neutrophils for monocytes/macrophages). IL-6 levels have been shown to be a key element in chronic inflammation. The expression of IL-6 is enhanced at the site of inflammation, and blocking the signaling of IL-6 has been shown to be effective in the prevention and treatment of inflammatory diseases (including arthritis and ulcers) [38]. Therefore, inhibiting compounds of IL-6 may be promising in the development of drugs for treating chronic diseases, as well as assisting in cancer therapy. Our results showed that the diterpenic acids 3-acetoxy-copalic (23.8% ± 8.2%), kaurenoic (11.2% ± 5.7%), kolavic-15-methyl ester (17.3% ± 4.2%), and copalic (4.2% ± 1.9%) at 50 mM inhibited the production of IL-6. Similar results were observed by Gelmini *et al.* [3] who demonstrated that a purified fraction of *C. langsdorffi* was able to promote the inhibition of IL-6 production about 28% to 37% using concentrations ranging from 0.1 mM to 1 mM. Lam *et al.* [39] also evaluated derivatives of acantoic acid, a diterpene, which was shown to be effective in suppressing the production of TNF-α and IL-6. Usually, labdane has a broad spectrum of biological activities previously reported, with potential anti-inflammatory activity. Giron *et al.* [34] also examined the effects of the natural diterpenes galeopsine, hispanolone and a series of nine derivatives of hispanolone. Two of these derivatives proved capable, at low concentrations (10 µM), of inhibiting NO^•^, TNF-α, and IL-6 production in RAW 264.7 macrophages stimulated with LPS.

Interleukin-10 (IL-10) is produced by activated macrophages and T lymphocytes and plays an important role in anti-inflammatory responses by inhibiting cytokine production in macrophages induced by lipopolysaccharide, including tumor necrosis factor- α, IL-6, and IL-12. The mechanism by which IL-10 inhibits cytokine production in macrophages remains unclear, though several models have been proposed [40]. Our results show that the kaurenoic diterpenes and 3-acetoxy-copalic induced the production of IL-10 at concentrations of 12.5 µM and 25 µM; however, this induction decreased at a concentration of 50 µM. The 3-hydroxy-copalic acid promoted the induction of IL-10 independent of the dose tested, and kolavic-15-methyl ester and hardwickiic acids did not induce the production of IL-10 (Figure 2C). 

Dehydroabietic acid is a derivative of abietic acid, an important diterpene component of the oleoresin fraction produced from coniferous species, and according to a study done by Kang *et al.* [41] this diterpene, at concentrations ranging from 20 µM to 50 µM, suppresses production of proinflammatory mediators such as MCP-1 and TNF- α in RAW 264.7 murine macrophages stimulated with LPS.

LPS promotes macrophage activation through its binding proteins CD14 and TLR4, which are present in the plasma membrane. Then, molecules involved in signal transduction are activated, such as the myeloid differentiation protein (MyD88), the receptor kinase of IL-1 (IRAK), the associated kinase TNF receptor (TRAF6)-inducing kinase NF-kB (NIK), and kappa β kinase (IKK). The activation of IKK promotes phosphorylation of the inhibitor of the transcription factor NF-kB (IKB- α), which favors polyubiquitination and subsequent degradation of IKB-α, which is located in the cytosol. Subsequently, NF-kB from the cytosol is released and translocated to the nucleus, where NF-kB (which is a ubiquitous transcription factor, since it regulates the transcription of several genes involved in immune and inflammatory responses) promotes the activation genes that encode proteins involved in the inflammatory response, such as TNF-α, IL-1β, IL-6, IL-10, among other nitric oxides [42].

The results of this study verify that none of diterpenes tested presented inhibitory effects on the production of TNF-α. Moreover, only the acids kolavic-15-methyl ester and kaurenoic inhibited IL-6 production, and may exert inhibitory effects on the production of acute phase proteins. While copalic, 3-hydroxy-copalic, 3-acetoxy-copalic, and kaurenoic acids can exert anti-inflammatory effects by induced IL-10 production, this cytokine also prevents tissue damage caused by bacterial and viral infections, as well as regulating and repressing the expression of pro-inflammatory cytokines. Thus, it can be concluded that the diterpenes can attenuate inhibition of inflammatory pathways mediated by transcription factor NF-kB, which in response can inhibit IL-6 production and increase IL-10 production.

## 3. Materials and Methods

### 3.1. Experimental Procedures 

NMR spectra were recorded on Varian INOVA 500 NMR spectrometers. The mass spectra were obtained using a gas chromatograph equipped with a Shimadzu QP-2010 mass spectra detector, using a VF-1MS capillary column (15 m × 0.25 mm id × 0.25 μm film thickness). The mass spectra were obtained with 70 eV.

### 3.2. Plant Material

All samples of oleoresin were kindly donated by other researchers, who primarily collected samples for botanical and ethnobotanical research about the genus *Copaifera*. These samples were collected in the Médio Juruá region of the state of Amazonas (see Newton *et al.* 2011 [43] for details) and in Ducke Reserve, in Manaus, also in the state of Amazonas. We used oleoresin from three identified species: *C. guianensis*, *C. langsdorffi*, and *C. multijuga*, as well samples of from unidentified *Copaifera* species. 

### 3.3. Extraction, Isolation and Structural Elucidation

The oleoresin of *Copaifera* spp*.* was fractionated by silica gel chromatography. This was evaluated using potassium hydroxide modified silica (SiO_2_-KOH) and was carried out with the material produced as follows: 160 g of silica gel (70–230 mesh) were stirred with 160 mL of a saturated solution of 10% KOH_aq_ for a period of 10 min, and were dried by hot air 24 h/80 °C. This mixture was then transferred into a glass column and washed with CH_2_Cl_2_. Crude oleoresin (8.0 g) was then applied to the top of this column and successively eluted, with 1200 mL of CH_2_Cl_2_ and 600 mL of MeOH, respectively. The MeOH fraction was concentrated to a quarter of the initial volume, acidified to pH 5 and extracted with CH_2_Cl_2_, which was then evaporated. The acidic fraction pH 5.0 was isolated with SiO_2_-KOH by flash chromatography.

The acid fraction of *C. multijuga* was subjected to columns, maintaining the ratio of 1:50 with silica gel (70–230 mesh) eluted with gradient solvent mixture of hexane and ethyl acetate, revealing the presence of three acids: copalic, 3-hydroxy-copalic, and 3-acetoxy-copalic. The acid fraction of *C. guianensis* was subjected to chromatographic column with the ratio of 1:50 copaíba oleoresin:silica gel (70–230 mesh) eluted with gradient solvent mixture of hexane and ethyl acetate, revealing the presence of the acid kolavic-15-methyl ester. The acid fraction of *C. langsdorffi* was subjected to column “flash”, according to Still *et al.* [44], with silica gel (70–230 mesh), eluting with isocratic solvent solution containing hexane, ethyl acetate and dichloromethane (7:2:1), revealing the presence of kaurenoic acid. The acid fraction of unidentified *C.* spp*.* was subjected to chromatographic column, as previously described, revealing the presence of hardwickiic acid.

**Figure 3:**
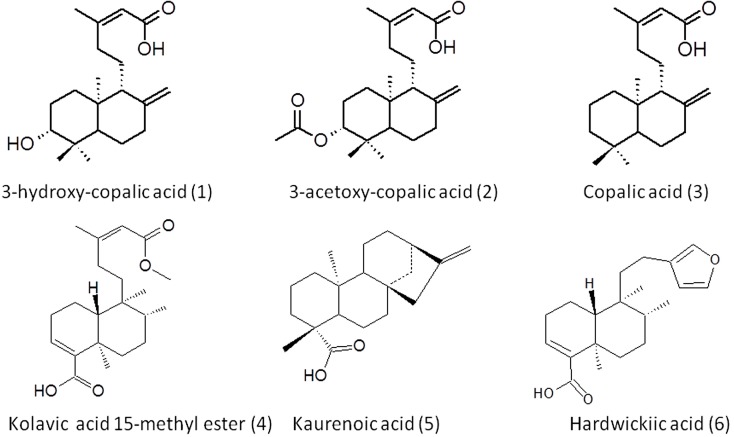
Chemical structures of diterpenes isolated from *Copaifera* sp.

All compounds were identified by the comparison of 500 MHz NMR spectra and IR with the data in the literature and the extensive analysis of 2D NMR involving ^13^C-^l^H COSY, HSQC and HMBC spectra. Figure 3 shows the molecular structure of the compounds.

### 3.4. Isolated Compounds Data

*3-Hydroxy-copalic acid* (**1**). White solid. ESI-MS: 319 [M], 301, 285 *m/z*; ^1^H-NMR (CDCl_3_) δ (ppm) *J* (Hz): 0.70 (s; 3H); 0.78 (s; 3H); 1.01 (s; 3H); 1.09 (dd; 12.45, 2.93; 1H); 1.17 (td; 13.18, 3.66; 1H); 1.35–1.45 (m; 1H); 1.51–1.60 (m; 3H); 1.61–1.68 (m; 2H); 1.70–1.75 (m; 2H); 1.76–1.80 (m; 2H); 1.93–2.03 (m; 2H); 2.17 (d; 1.10; 3H); 2.27–2.37 (m; 1H); 2.38–2.45 (m; 1H); 3.27 (dd; 11.72, 4.39; 1H); 4.52 (s; 1H); 4.83–4.90 (m; 1H); 5.68 (d; 1.10; 1H). ^13^C-NMR (CDCl_3_) δ (ppm) *J* (Hz):37.05 (C-1); 27.86 (C-2); 78.78 (C-3); 39.12 (C-4); 54.57 (C-5); 23.98 (C-6); 38.09 (C-7); 147.59 (C-8); 55.82 (C-9); 39.39 (C-10); 21.61 (C-11); 39.94 (C-12); 163.66 (C-13); 114.81 (C-14); 171.14 (C-15); 19.18 (C-16); 14.47 (C-17); 28.27 (C-18); 15.39 (C-19); 106.8 (C-20). 

*3-Acetoxy-copalic acid* (**2**). White solid. ESI-MS: 361 [M], 319, 301, 285 *m/z*; ^1^H-NMR (CDCl_3_) δ (ppm) *J* (Hz): 0.72 (s; 3H); 0.81–0.90 (m, 6H); 0.90–0.99 (m; 1H); 1.22–1.29 (m; 1H); 1.51–1.60 (m; 2H); 1.61–1.69 (m; 2H); 1.72–1.82 (m; 3H); 1.95–2.03 (m; 2H); 2.06 (s; 3H); 2.12–2.22 (m; 3H); 2.27–2.37 (m; 1H); 2.38–2.47 (m; 1H); 4.51–4.61 (m; 2H); 4.84–4.93 (m; 1H); 5.63–5.73 (m; 1H). ^13^C-NMR (CDCl_3_) δ (ppm) *J* (Hz):36.7 (C-1); 24.24 (C-2); 80.63 (C-3); 37.96 (C-4); 54.67 (C-5); 23.8 (C-6); 38.02 (C-7); 147.39 (C-8); 55.78 (C-9); 39.25 (C-10); 21.89 (C-11); 39.93 (C-12); 163.55 (C-13); 114.85 (C-14); 171.55 (C-15); 19.19 (C-16); 14.53 (C-17); 28.22 (C-18); 16.52 (C-19); 106.95 (C-20); 21.51 (C-21); 171.17 (C-22).

*Copalic acid* (**3**). ESI-MS: 303 [M], 285, 259, 99 *m/z*; ^1^H-NMR (CDCl_3_) δ (ppm) *J* (Hz):0.69 (s; 2H); 0.81 (s; 3H); 0.88 (s; 3H); 0.98–1.07 (m; 1H); 1.10 (dd; 12.45, 2.93; 1H); 1.15–1.23 (m; 1H); 1.26–1.35 (m; 2H); 1.36–1.43 (m; 1H); 1.49–1.55 (m; 2H); 1.56–1.61 (m; 2H); 1.67–1.77 (m; 3H); 1.94–2.04 (m; 2H); 2.18 (d; 1.10; 3H); 2.29–2.36 (m; 1H); 2.37–2.42 (m; 1H); 4.45–4.54 (m; 1H); 4.86 (d; 1.46; 1H); 5.31 (s; 1H); 5.68 (d; 1.46; 1H). ^13^C-NMR (CDCl_3_) δ (ppm) *J* (Hz):40.09 (C-1); 19.37 (C-2); 42.11 (C-3); 33.58 (C-4); 55.5 (C-5); 24.44 (C-6); 38.29 (C-7); 148.28 (C-8); 56.16 (C-9); 39.71 (C-10); 21.51 (C-11); 39.07 (C-12); 164.08 (C-13); 114.74 (C-14); 171.85 (C-15); 19.22 (C-16); 106.3 (C-17); 33.58 (C-18); 21.71 (C-19); 14.46 (C-20). 

*Kolavic acid-15-metyl ester* (**4**). ESI-MS: 347 [M], 315, 303, 271 *m/z*; ^1^H-NMR (CDCl_3_) δ (ppm) *J* (Hz): 0.74–0.84 (m, 6 H); 1.10–1.19 (m; 2H); 1.25 (s; 3H); 1.29 (d; 2.93; 1H); 1.37–1.45 (m; 2H); 1.46–1.51 (m; 1H); 1.60 (d; 5.86; 1H); 1.86 (d; 9.15; 1H); 1.99–2.08 (m; 3H); 2.19 (d; 1.46 Hz, 4 H); 2.27 (br. s.; 1H); 2.37 (d; 4.03; 1H); 2.68–2.78 (m; 1H); 3.70 (s; 3H); 5.69 (s; 1H); 6.82 (t; 3.84; 1H). ^13^C-NMR (CDCl3) δ (ppm) *J* (Hz): 16.82 (C-1); 28.61 (C-2); 142.24 (C-3); 137.53 (C-4); 40.32 (C-5); 36.21 (C-6); 24.38 (C-7); 37.84 (C-8); 36.32 (C-9); 45.46 (C-10); 36.79 (C-11); 34.46 (C-12); 161.39 (C-13); 114.95 (C-14); 172.82 (C-15); 19.16 (C-16); 15.87 (C-17); 167.24 (C-18); 18 (C-19); 33.38 (C-20); 50.8 (C-21).

*Kaur-9-en-16-oic acid* kaurenoic acid (**5**). White powder. ESI-MS: 301 [M] *m/z*; ^1^H-NMR (CDCl_3_) δ (ppm) *J* (Hz): 0.06–0.03 (m; 1H); 0.70–0.78 (m; 1H);0.86–0.92 (m; 2H); 0.88 (s; 2H); 0.93–1.02 (m; 2H); 1.07 (dd; 11.23, 5.37; 1H); 1.12–1.22 (m; 3H); 1.17 (s; 3H); 1.34–1.42 (m; 3H); 1.47–1.55 (m; 3H); 1.75–1.83 (m; 3H); 1.90–2.00 (m; 2H); 2.03–2.13 (m; 1H); 2.51–2.61 (m; 1H); 4.67 (s; 1H); 4.73 (br. s.; 1H). ^13^C-NMR (CDCl_3_) δ (ppm) *J* (Hz): 41.28 (C-1); 19.09 (C-2); 37.82 (C-3); 44.23 (C-4); 55.12 (C-5); 21.83 (C-6); 39.70 (C-7); 18.43 (C-8); 55.12 (C-9); 39.7 (C-10); 18.43 (C-11); 33.1 (C-12); 43.86 (C-13); 40.71 (C-14); 48.97 (C-15); 155.88 (C-16); 102.98 (C-17); 28.96 (C-18); 183.93 (C-19); 15.6 (C-20).

*Hardwickiic acid* (**6**). Yellow solid. ESI-MS: 315 [M], 271, 255, 223 *m/z*. ^1^H-NMR (CDCl_3_) δ (ppm) *J* (Hz): 0.76–0.79 (m; 2H); 0.80–0.86 (m; 3H); 0.88 (s; 1H); 0.97–1.06 (m; 1H); 1.15–1.22 (m; 1H); 1.23–1.28 (m; 3H); 1.40–1.42 (m; 1H); 1.44–1.46 (m; 1H); 1.47–1.55 (m; 3H); 1.56–1.60 (m; 2H); 1.64–1.73 (m; 2H); 2.15–2.25 (m; 2H); 2.17 (d; 1.53; 2H); 2.30–2.39 (m; 2H); 2.40–2.46 (m; 1H); 6.80–6.90 (m; 1H). ^13^C-NMR (CDCl3) δ (ppm) *J* (Hz): 17.45 (C-1); 27.28 (C-2); 140.3 (C-3); 141.46 (C-4); 37.59 (C-5); 35.82 (C-6); 27.49 (C-7); 36.25 (C-8); 38.82 (C-9); 46.69 (C-10); 38.64 (C-11); 18.18 (C-12); 125.58 (C-13); 110.97 (C-14); 142.7 (C-15); 138.38 (C-16); 15.96 (C-17); 172.45 (C-18); 20.54 (C-19); 18.3 (C-20). 

### 3.5. Cell Culture

The murine macrophage cell line J774 was kindly provided by Dr Leda Quercia Vieira (Laboratory of Nutrition and Genotobiology, UFMG, MG, Brazil) and were cultured at 37 °C in a humidified incubator with 5% CO_2_ in RPMI-1640 medium containing 10% fetal bovine serum (FBS), 50 U/mL penicillin and 50 μg/mL streptomycin (Invitrogen, Carlsbad, CA, USA). Lipopolysaccharide (LPS) was prepared as a 1 mg/mL stock solution in sterile water and stored at −20 °C. The diterpene compounds were added along with treatment with LPS.

**Cytotoxicity assay.** The cytotoxicity of diterpene compounds to the murine macrophage cell line J774 was determined by the Alamar Blue method as described by Nakayama *et al.* [45]. Briefly, adherent cells (5 × 10^3^ cells/well) were grown in 96-well tissue culture plates and exposed to substances (2.5 to 10 µg/mL) for 24, 48 and 72 h. After incubation, the Alamar Blue solution (10 μL of 0.4% Alamar Blue (resazurin) in PBS) was added and the cells were incubated for 3 h at 37 °C. Fluorescence was measured (excitation at 545 nm and emission at 595 nm) and expressed as a percentage of the cells in the control after background fluorescence was subtracted. As a positive control of cell death, doxorubicin (5 µg/mL) was used. The assays were performed in triplicate. For the assays with tumoral cells, an Alamar Blue assay was performed using the following cell lines: AGP01—gastric; HCT116—colorectal; MCF07—breast cancer; NIHOVCAR—ovary; SKAMELL4—melanoma; SF295—human glioblastoma, which were seeded in 96-well plates (0.5 × 10^4^ cells per well) and incubated in a CO_2_ atmosphere at 5% and 37 °C. After 24 h, diterpenes at 20 µM were added to each well and incubated for 72 h. Doxorubicin (10 μM) was used as a positive control and DMSO (0.1%) as a negative control. Before the end of the incubation, 10 µL of Alamar Blue (0.02%) was added to each well. The fluorescent signal was monitored using a multiplate reader using a 530–560 nm excitation wavelength and 590 nm emission wavelength. The fluorescent signal generated from the assay was proportional to the number of live cells in the sample, according to the manufacturer. The IC_50_ values and their 95% confidence intervals were obtained by nonlinear regression using the GRAPHPAD program.

### 3.6. Hemolysis Test

Blood was collected in heparinized-tubes from Wistar rats, and washed three times with saline solution (NaCl 0.85% + CaCl_2_ 10 mM) by centrifugation at 700 g for 10 min. The pellet of erythrocyte was re-suspended in saline solution for preparation of erythrocyte suspension −2% (SE). A total of 100 µL of the samples of diluted diterpenes was added to 96-well microplates in different concentrations (6.25 to 800 µM); 100 µL of SE was then added; and the samples were incubated for 60 min at 37 °C under constant agitation. The release of hemoglobin was determined after centrifugation (700 *g* for 10 min) by photometric analysis of the supernatant at 540 nm. Complete hemolysis was achieved using 0.2% Triton X-100 yielding the 100% control value. All procedures used during the experiments were maintained in accordance with internationally accepted principles for laboratory animal use and the experimental protocols were approved (n° 066/2011) by the Ethical Committee of the Animal Research of the Federal University of Amazonas, Manaus, Brazil.

### 3.7. NO^•^ Production Assay

NO**^•^** production by J774 cells was assayed by measuring the accumulation of nitrite in the culture medium using a Griess reaction [46]. Briefly, after incubation of the cells (1 × 10^6^ cells/mL) with diterpene compounds in different concentrations from 6.25 to 100 μM, cells were incubated for 24 h with LPS (1 μg/mL), at 37 °C in a 5% CO_2_ incubator. Nitric oxide was measured as NO_2_ in culture supernatant by reaction with Griess reagent. Absorbance of the reaction product was determined at 560 nm using a microplate reader (DTX 800, Beckman). Sodium nitrite was used as a standard to calculate nitrite concentrations.

### 3.8. Measurement of Cytokines

Macrophage cells (1 × 10^6^ cells/mL) were incubated with different concentrations of diterpene compounds (12.5 to 50 μM), and then stimulated with 1 μg/mL of LPS. The culture supernatants were collected after 24 h of LPS stimulation. The levels of cytokines in the culture media were measured by flow cytometry (BD Cytometric Bead Array—CBA—Mouse Inflammation kit) according to the manufacturer’s instructions.

### 3.9. Statistical Analysis

Results are expressed as the means and standard deviations of triplicate measurements. Each experiment was performed at least three times. Differences between groups were assessed by one-way analysis of variance (ANOVA) followed by the Tukey *post hoc* test. A value of *p* < 0.05 indicated significance.

## 4. Conclusions

In this study, we obtained six diterpenic acids, three labdanes (copalic acid, 3-hydroxy-copalic, and 3-acetoxy-copalic), two clerodanes (hardwickiic acid and kolavic acid 15-methyl ester), and one kaurane (kaurenoic acid) from *Copaifera* spp. oleoresin. These isolated compounds did not show toxicity to normal or tumor cell lines. The compound 3-hydroxy-copalic acid showed higher inhibitory activity for tyrosinase enzyme and NO^•^ production in LPS stimulated macrophages. Kolavic acid 15-methyl ester showed high lipoxigenase inhibition activity when compared to standard quercertin. Kaurenoic, 3-acetoxy-copalic, kolavic-15-methyl ester and copalic acids inhibited the production of IL-6 and kaurenoic, 3-hydroxy-copalic and copalic acids induced production of interleukin-10. Thus, we conclude that *Copaifera* spp. oleoresins contain components such as diterpenic acids, which are responsible for contributing to anti-inflammatory effects and other biological activities. However, the development of pharmaceutical formulations of copaíba oleoresin are needed in order to perform clinical trials aimed at demonstrating its application in the treatment of acute skin lesions and/or chronic inflammatory diseases.
